# Interactions between Multiple Recruitment Drivers: Post-Settlement Predation Mortality and Flow-Mediated Recruitment

**DOI:** 10.1371/journal.pone.0035096

**Published:** 2012-04-06

**Authors:** Antony M. Knights, Louise B. Firth, Keith Walters

**Affiliations:** 1 Department of Marine Science, Coastal Carolina University, Conway, South Carolina, United States of America; 2 School of Environmental Sciences, University of Liverpool, Liverpool, United Kingdom; 3 Department of Zoology, Ryan Institute, National University of Ireland Galway, Galway, Ireland; University of Maribor, Slovenia

## Abstract

**Background:**

Dispersal is a primary driver in shaping the future distribution of species in both terrestrial and marine systems. Physical transport by advection can regulate the distance travelled and rate of propagule supply to a habitat but post-settlement processes such as predation can decouple supply from recruitment. The effect of flow-mediated recruitment and predation on the recruitment success of an intertidal species, the eastern oyster Crassostrea virginica was evaluated in two-replicated field experiments. Two key crab species were manipulated to test predator identity effects on oyster mortality.

**Findings:**

Recruitment was ∼58% higher in high flow compared to low flow, but predation masked those differences. Predation mortality was primarily attributed to the blue crab Callinectes sapidus, whilst the mud crab Panopeus herbstii had no effect on recruit mortality. Recruit mortality from predation was high when recruit densities were high, but when recruit density was low, predation effects were not seen. Under high recruitment (supply), predation determined maximum population size and in low flow environments, recruitment success is likely determined by a combination of recruitment and resource limitation but not predation.

**Conclusions:**

Four processes are demonstrated: (1) Increases in flow rate positively affect recruitment success; (2) In high flow (recruitment) environments, resource availability is less important than predation; (3) predation is an important source of recruit mortality, but is dependent upon recruit density; and (4) recruitment and/or resource limitation is likely a major driver of population structure and functioning, modifying the interaction between predators and prey. Simultaneous testing of flow-mediated recruitment and predation was required to differentiate between the role of each process in determining population size. Our results reinforce the importance of propagule pressure, predation and post-settlement mortality as important determinants of population growth and persistence, but demonstrate that they should not be considered mutually exclusive.

## Introduction

Dispersal is a primary driver in shaping the future distribution of species in both terrestrial and marine systems [1], [2]. Connectivity among extant populations or expansion in range is fundamental to the persistence and demographic structure of a species [2] and reduces the risk of extinction under changing environmental conditions [3]. Despite the potential for long-distance dispersal, physical and biological barriers can limit realized dispersion to relatively short distances from the natal patch [4]. Physical barriers are perhaps the most obvious limit to dispersal with habitat fragmentation, climate change and anthropogenic disturbance each contributing to climbing global extinction rates [5]–[7]. Biological barriers can also play an important role in determining patterns in nature [8], not only affecting the dispersive propagule but also the sedentary adult life-stages common to many marine organisms. For example, during larval dispersion, mortality can exceed 90% [9] and reduce the larval pool to a population size which is ineffective [10]. Moreover, active ‘behavior’ such as vertical migration also may limit dispersion over large spatial scales (km) in addition to affecting recruitment patterns at small (cm to m) spatial scales [11], [12].

The roles of independent biological processes such as propagule supply, competition, and predation in determining population and community structure is relatively well studied and understood. However, an understanding of the potential interactions among biological processes and between biological and physical processes (e.g., fluid dynamics) is more limited [13]. For example, physical transport by advection can regulate not only the distance travelled by a propagule and the rate at which propagules are supplied to a habitat, but also settlement success [14], [15]. Localized phenomena such as turbulent mixing and bed shear increase with flow velocity and lead to substrate resuspension, reductions in substrate contact time by settling larvae, and reduced settlement success (e.g. [15]). Although the physical mechanisms of passive larval transport generally are well described, there is a decoupling of dispersal and population structure [16] most likely by post-settlement mortality processes, such as competition and predation [17].

Establishment or persistence of a population is dependent upon recruitment rates falling within thresholds at which density-dependent mortality does not preclude persistence and recruits arrive in sufficient abundance to dominate local interactions [18], [19]. However, linking the rate of supply, settlement and successful recruitment can be complicated by non-linear predation rates that vary in response to recruit density. Holling [20] demonstrated that changes in predator distribution, abundance (numerical) and feeding behaviors (functional) can change in response to changes in prey abundance. More recently meta-analyses of the response of communities to predators in multiple ecosystems have shown predation effects can vary considerably among systems and lead to cascading effects through the food web [21], [22].

A limited understanding of interaction effects on population dynamics (e.g. [23], [24]) combined with the recognition that system complexity continues to prevent accurate population predictions has led to a shift in focus toward simultaneous evaluation of multiple processes [25]. Larval supply, competition, and predation, either solely or in combination, have the potential to decouple dispersal from recruitment, and evaluation of these mechanisms continues to present a fundamental challenge in population connectivity studies [16]. Here, we experimentally tested the independent and combined effects of flow-mediated changes in recruit density and post-settlement mortality on the survival and abundance of an intertidal species. Recruitment is a critical process by which intertidal communities develop [19]; thus evaluation of how recruitment varies in response to changes in flow and mortality is critical in predicting population persistence. Using an important habitat-forming species in the USA, the eastern oyster *C. virginica,* two field experiments addressed the following questions: (1) does flow velocity affect the rate of recruitment; (2) does recruit density affect the likelihood of survival; (3) can post-settlement mortality be attributed to recruit density and if so, are mortality effects spatially consistent; (4) does recruit density affect the likelihood of predation mortality; and (5) does the identity of the predator matter?

## Methods

### Study Sites

**Figure 1:**
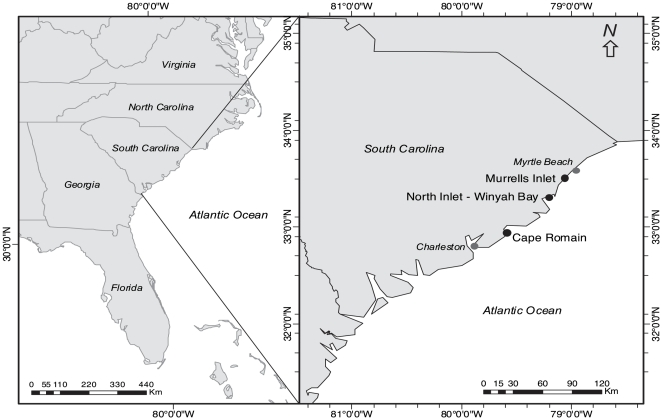
Study locations (bold) on the eastern coast of the USA.

The study was conducted at three locations along the coastline of South Carolina, USA: Murrells Inlet (33°33′ N, 79°01′ W); North Inlet (33°20′ N, 79°10′ W); and Cape Romain (32°55′ N, 79°38′ W) (Fig. 1). All three locations are estuarine, situated between the mainland and barrier islands and comprise salt marshes, mudflats and extensive intertidal oyster beds (*C. virginica*). Murrells Inlet is a commercially developed area, regularly visited by recreational fishers and boaters. North Inlet is a National Estuarine Research Reserve Site (NERRS) encompassing 50 km^2^ of tidal marsh and wetland and managed by the University of South Carolina (Baruch Marine Laboratory). Cape Romain is a Class-I wilderness national wildlife refuge covering 259 km^2^ and is a migratory bird refuge managed by the US Fish and Wildlife Service. All three locations are ocean-dominated, primarily accessible by boat, and consist of extensive tidal creek systems. Our experiment was conducted within and adjacent to large, extent native oyster beds on intertidal mudflats grading naturally from subtidal mudflat to low intertidal reef elevations. Permission to harvest oysters was provided under license from the South Carolina Department of Natural Resources (SCDNR) and the handling of all animals was conducted according to relevant national and international guidelines. The University of South Carolina (USC) and the US Fish and Wildlife Service (USFWS) kindly gave access to North Inlet and Cape Romain.

### Pilot Study 1: Flow Velocity Characterization of Sites

At each of the three locations, four sites were chosen at random to represent areas characteristic of ‘high’ and ‘low’ flow velocities (i.e. two high flow and two low flow sites per location). Site selection was based on observations and the flow velocity (e.g. cm/s) at each site was unknown. To test our categorization of sites within each flow category, the average flow velocity was estimated using gypsum (clod) dissolution [26]. Gypsum clods were mounted onto a ceramic tile (Versatile, USA). At each site (a total of 12 sites), 6 tiles were haphazardly placed and secured to the substrate by a cable tie attached to a polyvinyl chloride (PVC) pipe (20 mm diameter) driven into the mud. Clods were left at each site for 48 hr before collection and then dried to a constant mass. Proportional dissolution was calculated as the percentage difference between the initial and final mass of clod [see 27].

To test if flow velocities differed among sites and locations, mean proportional dissolution rates were compared using a 2-factor ANOVA with the factors: location (3 levels [Murrells Inlet; North Inlet; Cape Romain], random) and site (4 levels [1]–[4]; [5]–[8]; [9]–[12], random, nested in location) (*n*  =  6 tiles per site; a total of 72 plots). Student-Newman-Keuls (SNK) procedure was used to make post hoc comparisons among significant terms.

**Figure 2:**
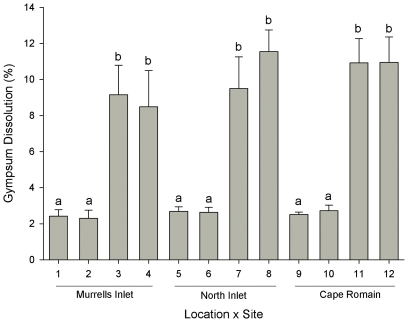
Flow velocity characterization of locations and sites using gypsum dissolution. Shown are mean proportional dissolution rates (± SD) of gypsum at each of three locations and four sites per location in South Carolina (*n*  =  6). Post hoc test outcomes are shown by letters (a, b) and indicate groups of means that are indistinguishable from each other (where letters differ p < 0.01).

Dissolution rates were significantly different between sites (Table 1, SNK tests, p < 0.001) but not different among locations. Post hoc tests revealed that dissolution at sites classified as high flow was ∼4x greater than those classified as low flow (Fig. 2, Table 1, SNK tests). There was no significant difference in dissolution between sites within each flow category (Table 1, Fig. 2, SNK tests, p < 0.01). Given that flow velocities (dissolution rates) at high or low flow sites were similarly represented (statistically) at each location, dissolution was ‘discretized’ [28] into two discrete, random orthogonal categories (here, ‘high flow’ or ‘low flow’).

**Table 1:** Flow velocity characterization of sites and locations by gypsum dissolution.

Source	df	MS	F	P
Location	2	0.52	0.03	0.97
Site (Location)	9	19.55	33.12	<0.001
Residual	60	0.59		

Cochran’s *C*  =  0.207, *ns.*

SNK test  =  << indicates p < 0.01.

Replicate gypsum dissolution clods (*n*  =  6) were haphazardly placed at each of four randomly chosen sites and three locations in South Carolina (see Figure 1 for locations). SNK outcomes are shown in Figure 2.

### Pilot Study 2: Effect of Caging on Flow

To test the effect of predation on recruitment success, experimental cages were used to prevent predators from accessing recruitment tiles. Cages can introduce experimental artifacts and affect recruitment by modifying flow rate [29]. Velocities can be reduced by up to 47%, but the use of a wire mesh with sufficiently wide diameter holes can minimize the likelihood of potential artifacts [29]. Cages were constructed from aluminum wire mesh (1 mm aperture) used to fully enclose each tile. To test the effect of the cage on water flow velocity, gypsum dissolution clods were again used [26]. Clods were placed on to tiles that were: (1) enclosed in a cage; (2) half enclosed by a cage (a procedural control, PC); or (3) had no cage (control). Clods were placed haphazardly throughout each site and left for 48 hr. Dissolution estimates were calculated as in Pilot study 1 above.

The effect of the cage was tested by comparing mean dissolution rates using a 2-factor ANOVA with the factors: flow (2 levels [High, Low], random) and treatment (3 levels [cage, PC, control], fixed, orthogonal) (Table 2). ‘Flow’ was included as a factor to test if cage effects varied under different flow conditions. Dissolution rates not differ between treatments (Table 2, Cage/PC/Control) and were not affected by differences in flow velocity (Table 2, interaction term, *ns*). This indicated that the holes in the mesh were sufficiently large to allow unrestricted water flow across the tile.

**Table 2:** Effect of cages of gypsum dissolution rate in high and low flow velocities.

Source	df	MS	F	P	SNK
Flow velocity	1	7.15	357.13	<0.001	Low << High
Cage/PC/Control	2	0.007	0.72	0.583	
Flow x Cage/PC/Control	2	0.009	0.47	0.628	
Residual	66	0.020			

Cochran’s *C*  =  0.347, *ns.*

Data are square root transformed.

SNK test  =  << indicates p < 0.01.

Proportional gypsum dissolution rates in cages, procedural control (PC) and control treatments in high and low flow velocities (*n*  =  12 gypsum dissolution clods per treatment per flow).

### Experiment 1: Effects of Flow Rate and Predation on Recruitment

To test the role of predation on recruit mortality, cages were used to exclude predators from recruitment tiles. Cages were designed to exclude most predators, in particular two crab species (the mud crab *Panopeus herbstii* and blue crab *Callinectes sapidus*) which have been previously identified as key predators of oyster [30], [31]. Unglazed red clay tiles (9 × 9 cm, Versatile, USA) provided a suitable substrate for oyster recruitment. Each tile was fully enclosed in a mesh cage (12 × 12 × 4 cm) constructed from aluminum mesh (1 mm aperture) to exclude predators. In the field, tiles were attached to PVC poles driven into the mud and inspected regularly to ensure the cages were maintained (i.e., the mesh remained clear of mud and fouling organisms). Twelve cages were haphazardly placed throughout each high and low flow site at each location (144 cages in total) (see Fig. 1 for locations). A further 12 uncaged (control) and 12 procedural control tiles were placed at each site to allow the effect of predation mortality on recruitment success to be determined. The procedural control for the cage treatment consisted of 1 mm mesh covering 50% of the tile and fully open to allow predators access to the tile. Tiles were collected after 14 wk and the abundance of living oyster spat (excluding scars) on all surfaces enumerated using a dissection microscope.

A 4-factor mixed model ANOVA was used to compare variation in recruit density using the factors: location (3 levels [Murrells Inlet; North Inlet; Cape Romain], random, orthogonal); flow (2 levels [High; Low], random, orthogonal); site (2 levels [A; B], random, nested in location and flow); and treatment (3 levels [Cage, Uncaged, Procedural control], fixed, orthogonal). In total, 432 plots were established across all locations.

### Experiment 2: Predation Mortality and Predator Identity Effects

To examine the effect of predator identity on recruit survival, oyster spat were exposed to *C. sapidus* and *P. herbstii* either independently or in combination (Table 3). Predator combinations were maintained using round cages (∼1 m diameter × 1 m height) and constructed of plastic mesh (1 mm aperture) attached to a frame of PVC piping (20 mm diameter). Cages were established at two sites within North Inlet, South Carolina (Fig. 1). In total, 30 plots were established comprising 5 treatments (including a control with no cage) (Table 3). Each combination was replicated (*n*  =  3) at each site. *C sapidus* and *P. herbstii* were placed in cages at densities of 1 and 4 per cage respectively and typical of the region (e.g. [32]; A.M. Knights *pers. obs.*).

**Table 3:** Experimental treatments testing the predation effects of two intertidal crab species on *Crassostrea virginica*.

Predator Combination	Identity of species included
A (+Blue +Mud)	*C. sapidus* and *P. herbstii*
B (+Mud)	*P. herbstii*
C (+Blue)	*C. sapidus*
D (−Blue −Mud)	None
E† (Control)	None

† Indicates no cage.

Treatment codes shown: +/− indicates species inclusion/exclusion; Blue crab *C. sapidus;* Mud crab *P. herbstii*; Control − uncaged treatment. Crab species were included at densities typical of the region (*C. sapidus -* 1 per cage; *P. herbstii* – 4 per cage) (A.M. Knights, *pers. obs.* and [32]).

Each cage was stocked with juvenile oysters sourced from a local hatchery. The shell length of each oyster (umbo to ventral edge) was measured to the nearest millimeter using vernier calipers (Mitutoyo, Japan). Oysters were marked with a small numbered waterproof tag attached to the shell using waterproof Sumo^TM^ glue (Loctite, USA). Ten oysters, ranging in size from 10–57 mm (mean size ± SE; 28.7 ± 7.5 mm) were randomly allocated to each treatment. The lengths of oysters allocated to each treatment were compared using ANOVA to test for homogeneous body size distributions throughout the treatments (Table S1). Oyster mortality (%) was recorded after 48 hr and predator identity effects were compared using ANOVA with the factors: Site (2 levels [A, B], random); *C. sapidus* (2 levels [present, absent], fixed, orthogonal); *P. herbstii* (2 levels [present, absent], fixed, orthogonal). A separate 1-factor analysis was used to compare control and predator combination treatments. Logistic regression was used [33] to determine if differences in predator identity (treatment) and oyster survival was dependent on the size of the prey.

### Statistical Analyses

GMAV5 was used for all ANOVA computations [34]. Prior to performing ANOVAs, Cochran’s test for homogeneity of variance was run and heterogeneous data were square-root transformed [35]. A Student–Newman–Keuls (SNK) procedure was used to make post hoc comparisons among levels of significant terms and an alpha significance level of 0.05 used in all analyses. The R-package was used for logistic regression computations [36].

## Results

### Experiment 1: Effects of Flow Rate and Mortality on Recruitment

**Figure 3:**
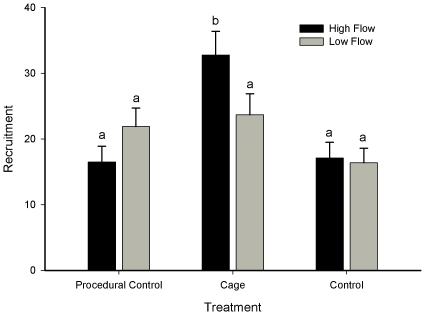
Mean recruitment (± SE) of *Crassostrea virginica* onto predator exclusion (cage), procedural control and open access (control) tiles. Recruitment tiles were established at two sites in each of three locations in South Carolina characterised by high and low flow regimes (*n* = 12; a total of 432 plots). Post hoc test outcomes are shown by letters (a, b) and indicate groups of means that are indistinguishable from each other (where letters differ p < 0.01).

Uncaged tiles suggested uniform recruitment at all locations irrespective of differences in flow velocity (Fig. 3, Table 4). However, caged tiles revealed that recruitment in high flow was ∼58% greater that at low flow locations (Fig. 3, Table 4) with densities of 33 ± 2.7 recruits per tile (4055 ± 338 m^−2^) and 21 ± 1.0 recruits per tile (2556 ± 123 m^−2^) respectively.

**Table 4:** *Crassostrea virginica* abundance on caged (C), uncaged (UC) and procedural control (PC) tiles at sites characterised by high and low flow velocities.

Source	Df	MS	F	P	SNK
Flow	1	231.15	0.11	0.77	
Location	2	1408.09	0.69	0.59	
Site (Flow x Location)	6	832.62	1.52	0.17	
Predation/Exclusion	2	5292.34	0.00	NO TEST	
Flow × Location	2	2037.71	2.45	0.17	
Flow × Predation/Exclusion	2	1912.41	11.20	0.02*	Low: (PC = UC = C)High: (PC = UC<<C)
Location × Predation/Exclusion	4	1027.09	6.02	0.06	
Predation/Exclusion × Site (Flow x Location)	12	457.47	0.83	0.62	
Flow × Location × Predation/Exclusion	4	170.74	0.37	0.82	
Residual	396	548.89			

Cochran’s test, *C*  =  0.08, *ns*.

SNK test  =  << indicates p < 0.01.

Replicate tiles (*n*  =  12) were established at two sites nested within 3 locations (Murrells Inlet, North Inlet, Cape Romain) and 2 flow regimes (high, low).

Predation effects also varied with flow. In high flow, recruitment on caged tiles was 52% greater than on uncaged treatments with 33 ± 2.7 and 17 ± 1.7 recruits per tile respectively (Fig. 3, Table 4, SNK tests, p < 0.05). In low flow, oyster densities were not significantly different between cage and uncaged treatments (Fig. 3, Table 4, SNK tests, *ns*) suggesting predation is dependent on oyster density. Recruitment on to procedural control tiles did not differ from uncaged tiles, indicating no artifact of the cage (Fig. 3, Table 4, SNK tests).

### Experiment 2: Predator Identity Effects on Recruit Mortality

**Figure 4:**
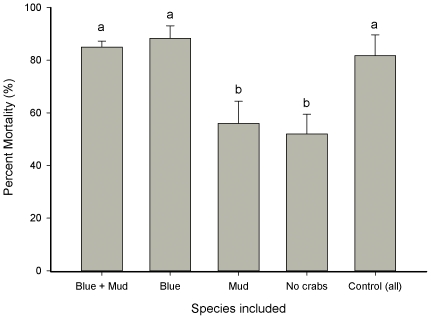
Predator identity effects on mortality of *Crassostrea virginica*. *C. sapidus* (blue) and *P. herbstii* (mud) were caged either independently or in combination with 10 oysters per cage. Crabs were included in cages at densities typical of those seen in the region (AM Knights, *pers. obs*. and [32]). Data are pooled from two sites at North Inlet (*n*  =  6, a total of 30 plots). Post hoc test outcomes are shown by letters (a, b) and indicate groups of means that are indistinguishable from each other (where letters differ p < 0.01).

Mortality from predation was significant but was species dependent. When *C. sapidus* was present, oyster mortality increased by ∼61% from 54 ± 2.8% to 87 ± 2.3% (Table 5, Fig. 4) but was unaffected by *P. herbstii* (Fig. 4, Table 5, SNK tests). Mortality did not change between cages including *C. sapidus* and *P. herbstii* (‘Blue + Mud’) or just *C. sapidus* (‘Blue’, Fig. 3) suggesting *P. herbstii* does not actively prey upon oysters within this size range. No significant difference in mortality between cage treatments containing *C. sapidus* and the control (open) suggests that *C. sapidus* may be the dominant predator in the system (Table 5, Fig. 4). Comparison of the size of dead or alive oysters indicated little evidence of prey size ‘preference’ by *C. sapidus* (see Table S1 and Fig. S1). When crabs were excluded, oyster mortality remained high (∼54%) but significantly lower than when *C. sapidus* was present (Fig. 4, Table 4, SNK tests).

**Table 5:** Proportional mortality of *Crassostrea virginica* in the presence or absence of *C. sapidus* (blue crab) and/or *P. herbstii* (mud crab) at two sites in South Carolina.

Source	df	MS	F	P	SNK
Site	1	42.67	0.15	0.70	
Blue crab (B)	1	6402.67	2401.00	0.01**	Not applicable
Mud crab (M)	1	0.67	1.00	0.05	
Blue crab x Mud crab	1	80.67	121.00	0.05*	−B+M = −B+M << +B+M = +B−M = C
Blue crab x Site	1	2.67	0.01	0.92	
Mud crab x Site	1	0.67	0.00	0.96	
Blue crab x Mud crab x Site	1	0.67	0.00	0.96	
Residual	16	4586.67			

Cochran’s test, *C*  =  0.27, *ns*.

** signifies p < 0.01; * signifies p < 0.05.

SNK test  =  << indicates p < 0.01; ‘−’ signifies species absent, ‘+’ signifies species present.

A separate 1-factor ANOVA was used to compare mortality between control (uncaged oysters) and the treatment containing both blue and mud crabs. No significant differences were found (*F*_1, 11_  =  0.16, *ns*).

## Discussion

Recruitment was greater in high flow sites, but this pattern was only evident when recruitment tiles were caged to exclude predators. When predators were absent, oyster mortality was generally high (∼54%) and increased when predators could access recruitment tiles. Predation was an important source of oyster mortality, but did not affect survival when prey densities were low. Testing the effect of two crab species on recruit mortality suggests that the blue crab *C. sapidus* is a major predator of the eastern oyster. In contrast, the mud crab *P. herbstii*, previously reported as an important predator of oysters [31], did not affect oyster survival in the current study.

Previous studies have demonstrated clear trends in population growth and abundance under varying rates of supply [18], [37]. The upper and lower thresholds of supply required for population growth are a balance between intra- and interspecific interactions, such as competition for resources (e.g. space and food) [18], [20], and predation [22]. It was hypothesized that increased flow velocities would increase supply. Physical transport processes are well known to affect propagule supply [38]. Increased flow (volume) can affect the rate of propagule delivery to a habitat [39], the distance travelled by larvae [14], or settlement success [14], [15], [40] and in previous studies, short-term recruitment has been used as a proxy for supply or settlement (e.g. [18], [20]). Here, uncaged tiles suggested that flow velocity had no effect on recruitment over the 14 wk period. However, a similar experiment conducted over shorter time scales (2 wk) at the same locations indicated increased flow increased short-term recruitment [20], but after 14 wk recruit densities were similar irrespective of flow rate and despite those short-term recruitment differences [20]. This indicated that post-settlement mortality rates must vary between high and low flow sites, to the extent that the differences in recruitment associated with changes in flow were masked. Mortality drivers were not differentiated although competition for resources (food) was cited as a potential mechanism [18]. Here caged tiles revealed a 58% increase in recruitment in high flow over low flow areas and suggested that predation has an important role in post-settlement mortality at high flow sites.

Larval supply is a pre-requisite for population growth and development [21] and the rate at which propagules are supplied to a habitat can affect establishment success [41], [42] by altering the interaction between recruits (prey) and their predators [43]. Simultaneous evaluation of different recruitment rates and predation revealed that when recruitment densities were high, predation was an important determinant of population size and perhaps more important than recruitment itself or other post-settlement mortality processes. This conclusion has also been reached in experiments in other marine (e.g. [44]) and terrestrial (e.g. [45]) habitats. Greater recruitment on caged tiles than uncaged tiles in high flow suggests that recruitment and/or resources are not limited in those areas [46]–[49] and do not account for the reduction in recruit densities as previously hypothesized [18]. If high flow sites were recruitment or resource limited, we would have expected recruit densities on caged tiles to be at levels similar to those on uncaged tiles. Although not explicitly tested here, a combination of density-independent mortality and competition for resources is likely to account for some post-settlement reduction in recruit density [50] but the magnitude of their effect did not appear as great as that of predation and/or their mortality mechanisms operate over shorter timescales than those used in this study. At low flow sites (low recruitment), no difference in recruit density between caged and uncaged tiles suggests that predation mortality in those areas is negligible. While larval supply and resource limitation were not directly tested, this suggests that propagule supply (the abundance of larvae, food particles or both) and not predation, is the limiting population-level process under low flow conditions [46]–[49].

Recruitment increased beyond ambient densities when predators were excluded at high flow sites, but was unaffected at low flow sites suggesting that predators responded to changes in recruit density. Changes in predator response to prey density have long been shown [20]. Mobile predators commonly adapt their foraging behavior such that they aggregate in areas of high prey density or increase their consumption rate in response to prey abundance [51], [52]. Either foraging mechanism could explain the disparities in observed recruitment patterns. However, the density of prey that is required to elicit a change in foraging behavior is poorly understood. For example, what prey density causes a predator to actively move into an area to forage? Contrasting the presence or absence of predation mortality and recruitment rate (as shown by caged tiles) suggests that oyster abundance must exceed a specific threshold (here, 18 oyster individuals per tile or a density of ≥ 2000 m^−2^) if predators are to change their foraging behavior. Densities exceeding this threshold were only recorded in high flow areas and in those areas, predation led to considerable recruit mortality (∼61%). In areas where densities were below the perceived threshold, predation effects were not apparent or the effect size insufficient to allow differences between caged and uncaged recruitment to be distinguished from inherent variation in recruitment.

An active response of predators to prey density seems a likely explanation for differences in predation effect between high and low flow sites, given both species are observed throughout the study areas (Knights, *pers. obs.*). Notably, there were no cases where recruits appeared to be consumed to the point of local extinction. In fact, recruit densities on uncaged tiles at high flow sites were markedly similar to densities recorded at low flow (recruitment) areas. This suggests that when recruit densities are below or become reduced below some density minima (in this case fewer than 2000 m^−2^), then the predator may become ‘uninterested’ in the prey resource and may move to an area where prey densities are likely to be greater. This sensitivity or detection of prey density may represent a behavioral response that reflects the need to increase foraging effort when prey are scarce [51] such that the energetic cost of foraging is reduced [30].

Tests of the independent and combined effects of the presence and absence of both predator species indicated that the presence of *C. sapidus* accounted for the majority of oyster mortality. In contrast, *P. herbstii* had little or no effect on recruit mortality. Excluding both crab species from oysters indicated that post-settlement mortality was generally high, on average ∼54%, and rising to over 80% in cases where *C. sapidus* was present. *C. sapidus* and *P. herbstii* are ubiquitous in saltmarshes of the eastern United States and co-exist within these areas [53]. Both species are known predators of intertidal invertebrates and *C. sapidus,* in particular, is known to be especially voracious, mobile and a highly adaptive predator [30], [31]. Therefore, the apparent absence of mortality due to *P. herbstii* is perhaps surprising. In an earlier experiment also comparing crab predation effects, treatments containing both *C. sapidus* and *P. herbstii* led to greater recruitment [53]. The authors concluded that the reduction in predation mortality was an indirect effect of interference competition between the two predator species. They argued that this competition resulted in negative consequences for both predator species and led to a release of prey from predation effects and thus, greater recruitment [53]. In the current study, predation mortality was unaffected by the combination of crab species within the cage suggesting no interference competition. Manipulating predator identity combinations within cages may not represent the scale at which the niches of these species overlap in nature (see [54] for review). However, given that rates of mortality in control cages were similar to those containing only the blue crab and lower in cages containing only mud crabs suggests no caging artifacts (i.e. artificially creating niche overlap) and therefore, the outcome is reflective of predation in intact (unmodified) communities.

Behavioral research suggests environmental conditions such as flow may have disproportionate effects on predation, particularly for those predators that use waterborne chemical odors to detect prey [55]. Environmental conditions can strongly affect how organisms perceive chemical signals from potential predators and prey [56], [57]. For example, *C. sapidus* can locate prey by following chemical odors, but this ability declines as flow velocity (and turbulence) increases [55], [58]. Several studies have implied a monotonic decline of foraging success with increasing flow velocity [52], [55], [59], however, a growing body of evidence suggests the relationship between foraging success and the effect of flow is not uniform [60]. This relationship may also vary by species. For example, the flow rate at high flow sites may have been insufficient to elicit a change in foraging behavior by *C. sapidus*, but sufficiently fast at both high and low flow sites to affect the response of *P. herbstii*. Neither here nor in many other studies is the effect of flow on chemical odor responses by predators and prey considered in terms of their effect on predator-prey interaction strength. However, given that local and/or regional differences in flow are likely, so then is a change in response by predator/prey to chemical cues and identification of regionally consistent patterns may be elusive when the effect of flow on predator-prey response(s) is not considered.

Our results provide a rigorous test of the combined fundamental effects of multiple recruitment drivers and demonstrate a non-linear response under different scenarios. Excluding predation demonstrated four processes. Firstly, that increases in flow rate positively affect recruitment success. Secondly, in high flow oyster reef environments at least, availability of resources (competition) is less important than predation. Thirdly, the comparison of caged and uncaged tiles demonstrates that predation is an important source of recruit mortality, but its effect is dependent upon recruit densities exceeding certain thresholds [43]. Fourthly, recruitment and/or resource limitation is likely a major driver of population structure and functioning, which modifies the strength of interaction between predators and their prey [46]–[49]. Appropriate tests designed to address several processes in combination were required to distinguish between their effects. For example, the effect of flow on recruitment would have been masked if predation had not been controlled. This highlights the need to include multiple processes in describing population and community structure and functioning and where possible, use empirical tests to quantify the role of those processes. This need has been recognised in several recent studies and is now viewed as critical to our understanding of ecological patterns and processes in both terrestrial and marine systems (e.g. [25], [61]). Our results reinforce the importance of propagule pressure, predation and post-settlement mortality as important determinants of population growth and persistence, but demonstrate that they should not be considered mutually exclusive.

## Supporting Information

Figure S1 **Comparison of the shell length (mean ± SD) of living and dead oysters in a cage containing one of five different predator combinations.** Predator(s) had access to 10 oysters and each combination was replicated (*n* = 3). Letters indicate the species included in the treatment (B  =  blue crab *Callinectes sapidus*; M  =  mud crab *Panopeus herbstii*) and ‘+/−’ signifies the presence or absence of the species in the cage.
(TIF)

Table S1 **Logistic regression comparing the shell length of living and dead oysters.** Oyster mortality estimated using five cage treatments containing varying predator identity combinations. + indicates predator inclusion, − indicates predator exclusion. *B*  =  *Callinectes sapidus; M*  =  *Panopeus herbstii; C*  =  *open access to all predators*. Each cage contained 10 oysters and each combination was replicated (*n*  =  3).
(DOC)
