# Supplementary figures and images for: Interactions between Multiple Recruitment Drivers: Post-Settlement Predation Mortality and Flow-Mediated Recruitment
Source: PLoS One. 2012 Apr 6;7(4):e35096. doi: 10.1371/journal.pone.0035096 (PMC3320868; doi:10.1371/journal.pone.0035096)

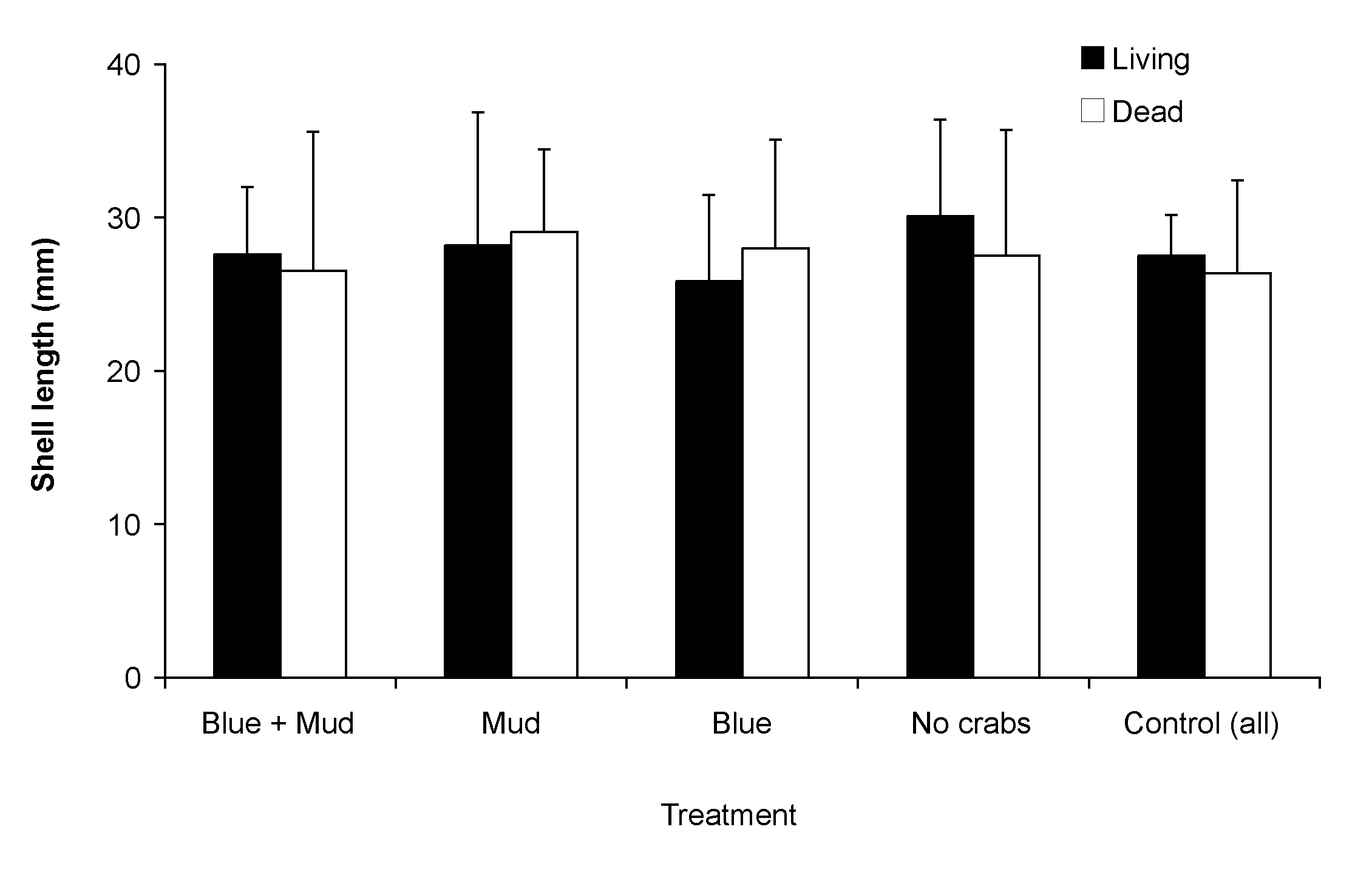

Supplement: Figure S1 — Comparison of the shell length (mean ± SD) of living and dead oysters in a cage containing one of five different predator combinations. Predator(s) had access to 10 oysters and each combination was replicated (n = 3). Letters indicate the species included in the treatment (B = blue crab Callinectes sapidus; M = mud crab Panopeus herbstii) and ‘+/−’ signifies the presence or absence of the species in the cage. (TIF) [file pone.0035096.s001.tif]
