# Supplementary material for: Interactions between Multiple Recruitment Drivers: Post-Settlement Predation Mortality and Flow-Mediated Recruitment
Source: PLoS One. 2012 Apr 6;7(4):e35096. doi: 10.1371/journal.pone.0035096 (PMC3320868; doi:10.1371/journal.pone.0035096)
Supplement: Table S1 — Logistic regression comparing the shell length of living and dead oysters. Oyster mortality estimated using five cage treatments containing varying predator identity combinations. + indicates predator inclusion, − indicates predator exclusion. B = Callinectes sapidus; M = Panopeus herbstii; C = open access to all predators. Each cage contained 10 oysters and each combination was replicated (n = 3). (DOC) [file pone.0035096.s002.doc]

**Antony M. Knights, Louise B. Firth, and Keith Walters. Interactions between multiple recruitment drivers: post-settlement predation mortality and flow-mediated recruitment.**

***Table S1. Logistic regression comparing the shell length of living and dead oysters.*** *Oyster mortality estimated using five cage treatments containing varying predator identity combinations. + indicates predator inclusion, - indicates predator exclusion. B = Callinectes sapidus; M = Panopeus herbstii; C = open access to all predators. Each cage contained 10 oysters and each combination was replicated (n = 3).*

| Coefficient | Estimate Std. | Error | *z* value | Pr (>|z|) |
| --- | --- | --- | --- | --- |
| Blue Crab + Mud crab (+B+M) | 2.01 | 1.61 | 1.25 | 0.21 |
| Shell length | -0.01 | 0.06 | -0.27 | 0.79 |
| Mud crab (+M) | 3.05 | 2.69 | 1.13 | 0.26 |
| Blue crab (+B) | -2.17 | 2.73 | -0.80 | 0.43 |
| No crabs (-B-M) | -0.25 | 2.47 | -0.10 | 0.92 |
| Control (C) | -4.33 | 3.46 | -1.25 | 0.21 |
| Shell length:+M | -0.12 | 0.09 | -1.42 | 0.16 |
| Shell length:+B | 0.07 | 0.10 | 0.73 | 0.47 |
| Shell length:-B-M | -0.02 | 0.08 | -0.22 | 0.82 |
| Shell length:C | 0.17 | 0.14 | 1.19 | 0.24 |

Dispersion parameter for binomial family taken to be 1

Null deviance: 167.6 on 149 *df*

Residual deviance: 155.2 on 140 *df*

AIC: 175.23
